# Virulence of an emerging pathogenic lineage of *Vibrio nigripulchritudo* is dependent on two plasmids

**DOI:** 10.1111/j.1462-2920.2010.02329.x

**Published:** 2010-09-06

**Authors:** Frédérique Le Roux, Yannick Labreuche, Brigid M Davis, Naeem Iqbal, Sophie Mangenot, Cyrille Goarant, Didier Mazel, Matthew K Waldor

**Affiliations:** 1 Laboratoire de Génétique et PathologieIFREMER, 3790 La Tremblade, France; 2 Channing Laboratory/Brigham and Women's Hospital and Harvard Medical School181 Longwood Ave, Boston, MA 02115, USA; 3 Département Lagons, Ecosystèmes et Aquaculture Durable en Nouvelle-CalédonieIFREMER, BP 2059, 98846 Nouméa Cedex, New Caledonia; 4 Institut Pasteur, Unité Plasticité du Génome Bactérien, Département Génomes et GénétiqueF-75015 Paris, France; 5 CNRSURA2171, F-75015 Paris, France; 6 CEA, Institut de GénomiqueGenoscope & CNRS UMR8030 Laboratoire de Génomique Comparative, Evry, France; 7 Institut PasteurNouméa, New Caledonia; 8 HHMIBoston, MA 02115, USA

## Abstract

Vibrioses are the predominant bacterial infections in marine shrimp farms. *Vibrio nigripulchritudo* is an emerging pathogen of the cultured shrimp *Litopenaeus stylirostris* in New Caledonia and other regions in the Indo-Pacific. The molecular determinants of *V. nigripulchritudo* pathogenicity are unknown; however, molecular epidemiological studies have revealed that recent pathogenic *V. nigripulchritudo* isolates from New Caledonia all cluster into a monophyletic clade and contain a small plasmid, pB1067. Here, we report that a large plasmid, pA1066 (247 kb), can also serve as a marker for virulent *V. nigripulchritudo*, and that an ancestral version of this plasmid was likely acquired prior to other virulence-linked markers. Additionally, we demonstrate that pA1066 is critical for the full virulence of *V. nigripulchritudo* in several newly developed experimental models of infection. Plasmid pB1067 also contributes to virulence; only strains containing both plasmids induced the highest level of shrimp mortality. Thus, it appears that these plasmids, which are absent from non-pathogenic isolates, may be driving forces, as well as markers, for the emergence of a pathogenic lineage of *V. nigripulchritudo*.

## Introduction

The family *Vibrionaceae* is comprised of a diverse group of organisms that reside within aquatic, mostly marine environments (Thompson *et al*., 2004). It encompasses the ancient and well-studied human pathogen, *Vibrio cholerae*, as well as some less thoroughly characterized human pathogens, including *Vibrio parahaemolyticus* and *V. vulnificus*. Perhaps less widely recognized are the consequences of vibrio infections in non-human species. Vibrios have been found to be pathogens of fish (*V. anguillarum*), coral (*V. shiloi*), shellfish (*V. splendidus*) and shrimp (*V. harveyi*, *V. penaeicida* and *V. nigripulchritudo*), and infections with these organisms have profound environmental and economic consequences (Goarant *et al*., 2004; Rosenberg and Falkovitz, 2004; Lopez and Crosa, 2007; Le Roux *et al*., 2009; Austin, 2010).

Vibrioses are the predominant bacterial infections in marine shrimp culture systems (Park *et al*., 1994; Ninawe and Selvin, 2009). In New Caledonia, where the cultivated penaeid shrimp *Litopenaeus stylirostris* is the second largest export, the industry has been affected since 1993 by a cold season vibriosis caused by *Vibrio penaeicida* (Syndrome 93; Goarant *et al*., 1999) that has largely prevented cultivation during this season. Additionally, since 1997, outbreaks during the summer period (‘Summer Syndrome’) have been caused by *V. nigripulchritudo* (Goarant *et al*., 2006b); the increasing prevalence of these epizootics threatens the viability of shrimp farming in this area. Mass mortalities of other penaeids (*Marsupenaeus japonicus* and *Penaeus monodon*) due to infection with *V. nigripulchritudo* have also been observed in Japan (Sakai *et al*., 2007) and in Madagascar (E. Chungue, pers. com.), suggesting that this organism potentially affects vast regions in the Indo-Pacific. Infections are characterized by an acute systemic vibriosis; however, little is known about the prevalence or diversity of pathogenic isolates of this organism or about the molecular basis of the disease that it causes.

In previous studies, *V. nigripulchritudo* isolated from a variety of sites in New Caledonia over an 8-year period have been characterized genetically and in an experimental model of infection (Goarant *et al*., 2006a; Goarant *et al*., 2006b). Multilocus sequence typing (MLST) revealed that all isolates from shrimp affected by Summer Syndrome, regardless of the year or site of isolation, clustered into a monophyletic clade. Studies of mortality after experimental infection suggested that pathogenicity is linked to this lineage. All isolates of the monophyletic cluster were demonstrated to be moderately pathogenic (20–80% mortality; MP) or highly pathogenic (80–100% mortality; HP) when injected into shrimp, while non-pathogenic isolates (NP, 0–20% mortality) were genetically diverse. MLST data did not permit discrimination between HP and MP isolates.

To further understand the differences between pathogenic and non-pathogenic isolates of *V. nigripulchritudo*, subtractive suppression hybridization (SSH; Winstanley, 2002) was performed using representative HP (SFn1) and NP (SFn118) isolates (Reynaud *et al*., 2008). DNA fragments isolated using this approach were subsequently scored, using macroarrays, for their presence in HP, MP and NP strains. This study led to the identification of 13 DNA fragments detectable in the HP isolates and absent from both NP and MP isolates. Of these, 10 corresponded to putative open reading frames (ORFs) harboured by a 11.2 kb plasmid, designated pB1067 (previously named pSFn1), raising the possibility that this element contributed to the emergence of HP strains (Reynaud *et al*., 2008). The study also identified three chromosome-derived fragments that were found in nearly all HP strains (which we have now localized to a genomic island of chromosome 2) and 55 fragments that were shared by HP and MP strains but absent from NP strains, any of which might encode factors that contribute to *V. nigripulchritudo* virulence. The subsequent and ongoing sequencing of the complete genome of the *V. nigripulchritudo* HP strain SFn1 (Genoscope, Evry, France) revealed that two of the 55 fragments, which contain sequences homologous to transposases, are encoded within a previously undetected large (247.2 kb) plasmid here named pA1066. This finding raised the possibility that loci within pA1066 might be important for the pathogenicity of *V. nigripulchritudo.*

In this study, we assessed the prevalence of pA1066 or related plasmids among pathogenic and non-pathogenic isolates and found that HP and MP strains always carry distinct forms of this replicon. The precise correlation between the presence of the HP form of pA1066, pB1067, and previously described HP-specific chromosomal loci suggests that these features are genetically linked. We also explored the contribution of the HP-specific plasmids to the virulence of these strains. By infecting shrimp with derivatives of an HP *V. nigripulchritudo* strain from which pA1066 and/or pB1067 have been lost we found that both plasmids are necessary for full virulence, particularly when using a bath challenge method of infection that mimics natural infection conditions. In addition, we found that supernatant toxicity is a characteristic of HP, but not MP, strains. HP strains release a large, heat-sensitive factor into culture supernatants that is toxic upon injection into shrimp, and the production and/or activity of this factor is dependent upon the presence of pA1066.

## Results

### The large plasmid pA1066 encodes a putative MARTX toxin and may be conjugative

Genome sequencing of *V. nigripulchritudo* strain SFn1 is in progress at the Genoscope (Evry, France). Although annotation of this genome is not yet completed, assembly of contigs revealed that SFn1 has the largest vibrio genome described to date, confirming observations previously made (Okada *et al*., 2005). It consists of two circular chromosomes of 4062 (chromosome 1) and 2214 (chromosome 2) kb and two plasmids of 11.7 (pB1067) and 247.2 (pA1066) kb, with an average per cent G+C content of 45.9%, 45.5%, 45.7% and 45.3% respectively. The plasmid pB1067 (Accession No.: NC_010733), which was previously described (Reynaud *et al*., 2008), contains 10 ORFs and encodes a putative partitioning protein, conjugal transfer repressor, peptidase S49, and seven conserved hypothetical proteins. Identification of the large plasmid, pA1066, in the SFn1 genome was unexpected, since this replicon had not been previously detected.

**Figure 1:**
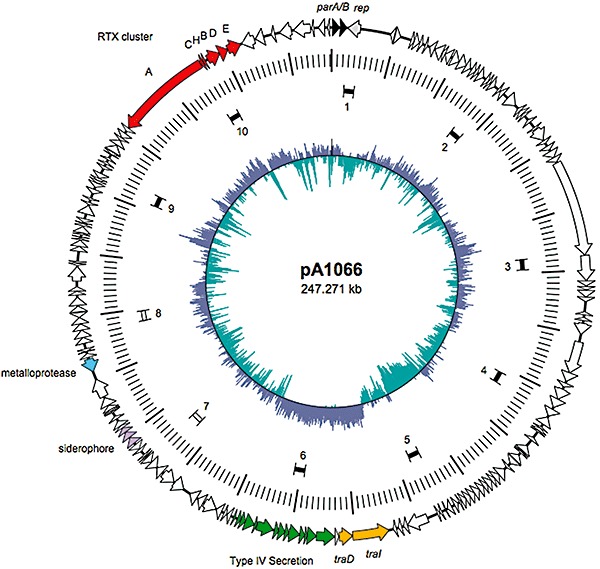
Circular representation of the *V. nigripulchritudo* pA1066 plasmid. From the outside inwards: the first circle shows pA1066 ORFs (in gray and black replication and partitioning; in green and orange conjugative transfer; in red MARTX cluster; in blue metalloprotease; in purple siderophore ABC transporter); the second circle shows the scale (small tick marks = 1 kb; large tick marks = 10 kb); the third circle shows the PCR products amplified for pA1066 detection and genotyping (filled symbols = amplified from HP and MP isolates; open symbols = amplified only from HP isolates); the fourth circle shows the GC per cent.

A total of 179 ORFs were predicted from the pA1066 plasmid sequence (Fig. 1). Nearly half of the pA1066 genes code for proteins of unknown function. Like many large plasmids, pA1066 appears to have a modular architecture, which is a likely consequence of evolutionary mosaicism (Fernandez-Lopez *et al*., 2006). Three notable putative functional modules include: a replication/segregation module (Fig. S1), a MARTX (multifunctional autoprocessing repeats in toxin) toxin cluster (Fig. S2A) and a cluster that appears to encode conjugation machinery (Fig. S3). The potential roles of additional genes with characterized homologues are annotated in the complete plasmid sequence (Accession No.: FP89324).

The putative Rep protein (VIBNI_0003) bears very limited homology to known proteins. However, it contains three short stretches of amino acids (6–15 residues) that are identical to sequences present in RctB, the *V. cholerae* Chr2 replication initiator. Furthermore, VIBNI_0003 is flanked by a region that exhibits features similar to those found in *V. cholerae oriC2*, including a DnaA box, 18 repeat sequences and an AT-rich segment (Fig. S1), all of which are important for RctB-based replication in *V. cholerae* (Egan and Waldor, 2003). These observations, coupled with the proximity of *parA/parB* homologues (VIBNI_001 and _002) which are often found near origins of replication (Bignell and Thomas, 2001), suggest that this region might comprise the replicative origin of this plasmid. In support of this hypothesis, we found that a functional replicon was generated by ligation of pA1066 DNA spanning this region (position 0–9.2 kb) to a Spec^R^ cassette (data not shown). Notably, this plasmid replicated in *V. cholerae* but not in *Escherichia coli*, as was also observed for some *rctB*-dependent replicons (Egan and Waldor, 2003).

A six-gene cluster (211–232.9 kb) in pA1066 that encodes a putative toxin (RtxA, VIBNI_0210), a putative acyltransferase (RtxC, VIBNI_0211), an uncharacterized protein (RtxH, VIBNI_0212) and a putative type I secretion system (RtxBDE, VIBNI_0213 to 15) shows an organization similar to MARTX toxin clusters (Fig. S2A). These clusters, which have been characterized primarily in *V. cholerae* and *V. vulnificus* but have also been found in numerous other organisms, enable secretion of large, typically multifunctional, protein exotoxins, which are encoded by *rtxA* (reviewed by Satchell, 2007). Close to the *rtxA* gene we identified a pseudogene homologous to a recombinase, suggesting that this cluster was acquired via horizontal gene transfer.

As in *V. cholerae* and *V. vulnificus*, the putative *V. nigripulchritudo* RtxA is extremely large (4990 amino acids; 536 kDa) and contains numerous modules (generously annotated by Dr Karla Satchell; Fig. S2B). It is predicted to contain the three domains found in all MARTX: large repeat regions at the N- and C-termini, and a cysteine protease domain that is essential for the autoprocessing and toxicity of MARTX in *V. cholerae* (Sheahan *et al*., 2007). In addition, it contains putative effector domains that have been identified in a subset of other MARTX toxins. These include a homologue of the Rho GTPase domain that has been associated with the ‘cell rounding’ effect caused by MARTX_Vc_ (Sheahan and Satchell, 2007), an ExoY adenylate cyclase domain, and two domains with unknown function, DUF1 and DUF3. The closest homologues for each domain are found within distinct organisms, suggesting that the pA1066 MARTX toxin, like other members of this family, is a mosaic of independently acquired effector domains (Satchell, 2007).

The pA1066 sequence also includes a gene cluster (114.7–140.7 kb; VIBNI_111 to 127) that may impart mobility to the plasmid. This cluster includes homologues of the core proteins of a type IV secretion system, the coupling protein TraD and a relaxase-helicase TraI (Fig. S3) (Lawley *et al*., 2003). The presence of these genes suggests that pA1066 may be a conjugative plasmid; however, since we have been unable to generate a marked version of this replicon (discussed below), it has not been possible to assess this experimentally. The average GC % of this region (54%) is clearly different from the whole plasmid (45.3%) and the region is flanked by two pseudogenes – a transposase and a helicase – suggesting that these sequences evolved separately from the remainder of the plasmid and were acquired via horizontal gene transfer.

### HP and MP strains contain distinct forms of pA1066

**Figure 2:**
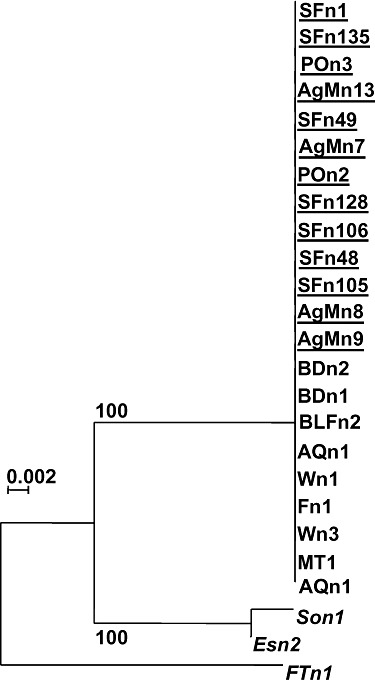
Phylogenetic tree of the pA1066 *rep* gene. The tree was built by the Neighbour-joining method based on sequences aligned using Seaview. Branch lengths are drawn to scale and are proportional to the number of nucleotide changes. Number at each node represents the percentage value given by bootstrap analysis of 1000 replicates. HP strains are in bold and underlined, MP in bold, NP in italic.

The finding that 2 of 55 HP/MP-specific fragments identified by SSH are encoded within pA1066 suggested that this large plasmid might be linked to virulence. Therefore we undertook a more systematic evaluation of the presence of this plasmid within our *V. nigripulchritudo* collection (Goarant *et al*., 2006b). Several attempts to detect pA1066 by pulse field gel analysis failed, apparently due to DNA degradation, which tends to be more extensive in DNA samples from *V. nigripulchritudo* than in those from other *Vibrio* species (data not shown). Consequently, we amplified 1500 bp fragment of the putative *rep* gene from this collection of isolates. This region was successfully amplified from all HP and MP strains tested and from 3 of 15 NP strains. Sequence analysis revealed that this region is identical within all HP and MP strains, while NP strains contain several distinct sequence variants. Thus, phylogenetic analysis of pA1066 *rep* (Fig. 2) is fully congruent with the previous phylogenetic analyses based on MLST data, in that both led to the grouping of MP/HP strains in a monophyletic group, whereas NP strains were found to be polyphyletic.

Since strains within this monophyletic cluster have distinct phenotypes in the experimental model of infection (i.e. some HP, some MP), we explored whether variability in regions of pA1066 other than its origin could be detected among these strains, which could potentially account for the range of phenotypes. For this analysis, we attempted to amplify 10 regions of 2 kb that are distributed at 25 kb intervals around pA1066 from each strain (Fig. 1, third circle). Each HP strain tested yielded a product of the expected size for 10/10 primer sets, suggesting that a pA1066-like plasmid (named type HP) is present in all these HP isolates (Table 1). In contrast, all MP isolates tested yielded a product of the expected size for only 8/10 primers sets (including MARTX). No product was obtained from MP strains for two primer sets (PCR 7, 8). Thus, it appears that MP strains consistently carry a different form of pA1066 (named pA1066 type MP) than the one present in HP strains. The proteins whose presence may be restricted to HP isolates include a putative siderophore ABC transporter and a putative metalloprotease, both of which are linked to virulence in other pathogenic vibrios (Le Roux *et al*., 2007; Lopez and Crosa, 2007). Alternatively, sequence polymorphisms may account for their failure to be amplified. Only 4 of 15 NP isolates yielded any PCR products in these analyses, and no NP strain yielded more than four products (Table 1). This result is consistent with our previous observation that pA1066 *rep* can rarely be amplified from NP strains, and suggests that any pA1066-like plasmid present in an NP isolate is only distantly related. Collectively, these results suggest that the presence of an HP-type pA1066 plasmid, like the previously demonstrated presence of pB1067, is a reliable marker of high pathogenicity in New Caledonian *V. nigripulchritudo.* Furthermore, the identity of the sequence of the *rep* gene in MP- and HP-type pA1066 plasmids suggests that these plasmids are derived from a common ancestor that has subsequently differentiated into the MP and HP types.

**Table 1:** PCR detection of pA1066 (PCR products are localized in Fig. 1) within the strain collection.

Virulence	Strain	1	2	3	4	5	6	7	8	9	10
HP	SFn1	+	+	+	+	+	+	+	+	+	+
	POn19	+	+	+	+	+	+	+	+	+	+
	SFn27	+	+	+	+	+	+	+	+	+	+
	SFn135	+	+	+	+	+	+	+	+	+	+
	SFn127	+	+	+	+	+	+	+	+	+	+
	AgMn13	+	+	+	+	+	+	+	+	+	+
	SFn49	+	+	+	+	+	+	+	+	+	+
	AgMn7	+	+	+	+	+	+	+	+	+	+
	POn2	+	+	+	+	+	+	+	+	+	+
	SFn128	+	+	+	+	+	+	+	+	+	+
	AgMn10	+	+	+	+	+	+	+	+	+	+
	SFn106	+	+	+	+	+	+	+	+	+	+
	SFn2	+	+	+	+	+	+	+	+	+	+
	AgMn12	+	+	+	+	+	+	+	+	+	+
	SFn105	+	+	+	+	+	+	+	+	+	+
	AgMn8	+	+	+	+	+	+	+	+	+	+
	AgMn9	+	+	+	+	+	+	+	+	+	+
MP	BDn2	+	+	+	+	+	+	−	−	+	+
	BDn1	+	+	+	+	+	+	−	−	+	+
	BLFn1	+	+	+	+	+	+	−	−	+	+
	BLFn2	+	+	+	+	+	+	−	−	+	+
	Wn13	+	+	+	+	+	+	−	−	+	+
	ENn2	+	+	+	+	+	+	−	−	+	+
	AQn1	+	+	+	+	+	+	−	−	+	+
	Wn1	+	+	+	+	+	+	−	−	+	+
	Fn1	+	+	+	+	+	+	−	−	+	+
	MT1	+	+	+	+	+	+	−	−	+	+
	AQn2	+	+	+	+	+	+	−	−	+	+
NP	FTn1	−	−	+	−	−	+	+	+	−	−
	POn13	−	−	+	−	−	+	−	−	−	−
	SOn1	−	−	−	−	−	+	−	−	−	−
	AgMn1	−	−	−	−	−	+	−	−	−	−
	SOn2	−	−	−	−	−	−	−	−	−	−
	ESn2	−	−	−	−	−	−	−	−	−	−
	ENn1	−	−	−	−	−	−	−	−	−	−
	POn10	−	−	−	−	−	−	−	−	−	−
	Fn2	−	−	−	−	−	−	−	−	−	−
	SFn115	−	−	−	−	−	−	−	−	−	−
	SVn3	−	−	−	−	−	−	−	−	−	−
	AgMn3	−	−	−	−	−	−	−	−	−	−
	POn12	−	−	−	−	−	−	−	−	−	−
	POn4	−	−	−	−	−	−	−	−	−	−
	SFn118	−	−	−	−	−	−	−	−	−	−

### Disruption and curing of HP-linked plasmids in *V. nigripulchritudo*

Given the correlation between the presence of pA1066, pB1067, and high pathogenicity, we wanted to assess the importance of either or both of these plasmids for *V. nigripulchritudo* virulence. We therefore attempted to disrupt potential virulence-linked ORFs in each replicon, by introducing a suicide vector, derived from the oriR6K-dependent vector pSW25T (Demarre *et al*., 2005), that would integrate within these loci. In addition, we attempted to cure each plasmid from the HP strain SFn1 by introducing an incompatible and selectable replicon. All of these genetic manipulations relied upon conjugation to introduce sequences into SFn1, as successful protocols for transformation of *V. nigripulchritudo* have not yet been established. Using this approach, we managed to disrupt numerous genes within pB1067 by insertional mutagenesis (Tables 2 and 3 and data not shown); however, strains in which a suicide vector had integrated within pA1066 could never be obtained. Subsequently, during characterization of the pB1067 mutants, we noted that all of these strains no longer contained pA1066 (data not shown). Similarly, when pSW110, which consists of the pB1067 origin of replication (see *Experimental procedures*) fused to pSW25T, was introduced into SFn1 in order to cure it of pB1067, pA1066 was also lost from the majority (9 of 10) of strains (data not shown). Collectively, these results suggest that the presence of pSW25T sequences, integrated either into another replicon (e.g. as in insertional disruption of pB1067) or within an independent replicon (e.g. as in pSW110), interferes with replication and/or maintenance of pA1066. The mechanism underlying the incompatibility of these sequences has not yet been determined. However, we found that conjugative transfer to SFn1 of a P15A-dependent plasmid that confers spectinomycin resistance yielded transconjugants that contained both pB1067 and pA1066 (not shown), suggesting that neither the resistance cassette nor the process of conjugal DNA transfer is likely to interfere with maintenance of pA1066. It seems therefore most likely that the R6K origin of replication in pSW25T interferes, in an unknown fashion, with pA1066, and that this accounts for the inability to introduce mutations within pA1066 noted above.

**Table 2:** Strains used and constructed in this study.

Name	Plasmid(s)
SFn1	pB1067, pA1066
VN110	pSW110
VN157	pA1066
VN68	pB1067::pSW68
VN120	pB1067::pSW120

**Table 3:** Plasmids used and constructed in this study.

Plasmid	Description	Reference
pSW25T	*oriV*_R6Kγ_; *oriT*_RP4_; [Spec^R^]	Demarre *et al*. (2005)
pSW110	pSW25T::(2700–3400)_pB1067_	This study
pSW68	pSW25T::(2000–2530)_pB1067_	This study
pSW120	pSW25T::(28–480)_pB1067_	This study

Fortunately, by removing antibiotic selection from the single SFn1 derivative that maintained pA1066 in the presence of pSW110, we were able to isolate a strain that contained only pA1066 (referred to as VN157). Subsequent transfer of a pB1067::pSW120 cointegrate into VN157 resulted in loss of pA1066, indicating that pA1066 within VN157 has not become routinely compatible with pSW25T and suggesting that this plasmid maintains its natural characteristics. The wild-type (wt) SFn1, VN157, the numerous strains containing only derivatives of pB1067, and VN110 (cured of both plasmids; contains only the pB1067 origin of replication fused to pSW25T) all displayed similar kinetics of growth and final absorbance at 600 nm in different media, indicating that plasmid curing did not have deleterious effects upon growth (not shown). The virulence of these strains was therefore assessed in experimental models of *V. nigripulchritudo* infection.

### Experimental challenge demonstrates that both plasmids are necessary for virulence

The previous study of *V. nigripulchritudo* pathogenicity that yielded the distinction between HP, MP and NP strains utilized an experimental infection model in which bacteria are intramuscularly injected into healthy juvenile shrimp (*L. stylirostris*). While this assay is fairly convenient and yields reproducible results, it is not particularly analogous to the natural route of infection. We therefore assayed the virulence of our mutants using three experimental models: shrimp intramuscularly injected with bacteria, shrimp transiently immersed into bacteria-contaminated waters, and shrimp intramuscularly injected with bacterial culture supernatants. Collectively, these experiments revealed that pA1066 and pB1067 are both required for full pathogenicity of *V. nigripulchritudo*, although their results differ as to whether the effects of these plasmids are independent or interdependent. In addition, they indicate that at least some of the virulence of *V. nigripulchritudo* may be due to pA1066-dependent release of a toxic compound.

**Figure 3:**
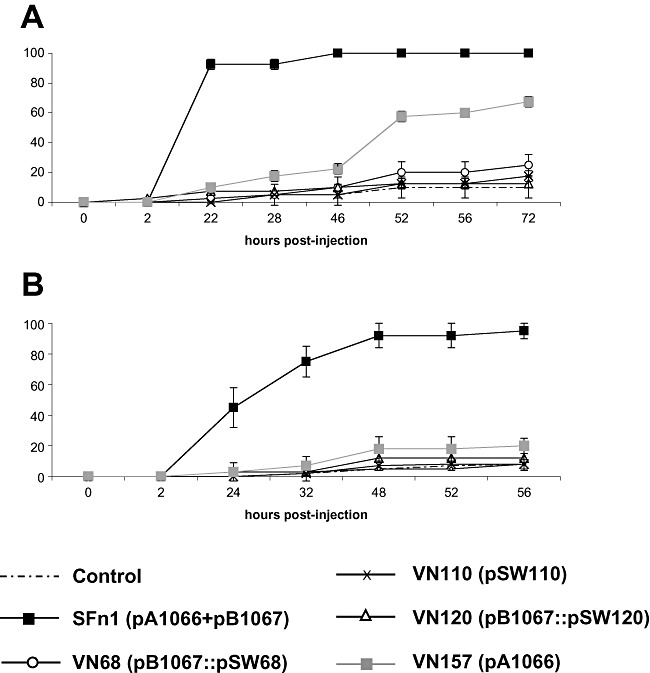
Shrimp mortality in response to experimental infection with *V. nigripulchritudo*. For the injection challenge (A), 50–500 cfu of the tested strain was intramuscularly injected into shrimp (*n* = 10–20, in duplicate). For the immersion challenge (B), shrimp (*n* = 10–20, in triplicate) were incubated 2 h with 10^5^ cfu ml^−1^ of *V. nigripulchritudo*, then transferred to 100 l tanks containing filtered seawater. Survival was monitored daily over a 3-day period.

As previously reported, intramuscular injection of the HP strain SFn1 into shrimp results in the death of > 80% of shrimp (typically 100%) within 24 h (Fig. 3A; Goarant *et al*., 2006b). In contrast, shrimp injected with VN68, VN120 and VN110, which lack pA1066 or both plasmids, displayed a maximum mortality of 20% even 3 days post-injection. This result suggests that pA1066 is crucial for the high pathogenicity phenotype of strain SFn1. Injection of VN157, which contains pA1066 but lacks pB1067, resulted in an intermediate phenotype. Approximately 70% of shrimp died, but the majority did not succumb to the infection until at least 2 days post-injection. Thus, pB1067 is not pivotal for virulence, but it does augment virulence in this model.

To more accurately model the natural process by which vibriosis develops in shrimp, we utilized an immersion model of infection (Saulnier *et al*., 2000). Shrimp were placed in filtered seawater contaminated with *V. nigripulchritudo* only transiently (2 h), then transferred to clean water for the duration of the experiment. Under these assay conditions, only *V. nigripulchritudo* containing both pA1066 and pB1067 induced significant mortality (> 80% by 48 h; Fig. 3B). Strains containing one or no plasmid induced < 20% mortality, even several days after infection. For the strains containing no plasmid this low mortality may result from a reduced ability to infect/colonize the host shrimp, as enumeration of *V. nigripulchritudo* in shrimp haemolymph 24 h after exposure revealed that their abundance was several orders of magnitude lower than that of wt SFn1 [SFn1 and VN157: ∼10^4^ colony-forming units (cfu) ml^−1^; VN68 and VN120: ∼10^3^ cfu ml^−1^; VN110: 0 cfu ml^−1^]. However, colonization of wt SFn1 and VN157 was equivalent, suggesting that colonization by *V. nigripulchritudo* is not sufficient to induce mortality in *L. stylirostris*; additional, presumably pB1067-dependent factors must also be necessary.

**Figure 4:**
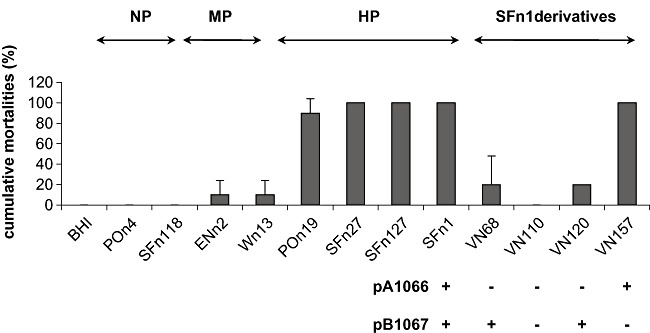
Shrimp mortality in response to injection of *V. nigripulchritudo* culture supernatants. Animals (*n* = 8–12) were injected with 100 μl of bacterial supernatant prepared from overnight cultures. Survival was assessed after 24 h. Experiments were conducted in duplicate.

Finally, we assessed whether products released by the various strains of *V. nigripulchritudo* into their surroundings were detrimental to *L. stylirostris. In vivo* injection of culture supernatants, extracellular products (ECP), or purified metalloproteases is a classic assay of bacterial exotoxicity (Goarant *et al*., 2000; Labreuche *et al*., 2006; Le Roux *et al*., 2007). We therefore tested the exotoxicity of a diverse panel of *V. nigripulchritudo* isolates, including strains previously classified as HP (*n* = 4), MP (*n* = 2) and NP (*n* = 2), in addition to testing supernatants derived from our set of SFn1 derivatives. Strikingly, 1 day post-injection, 10% and 0% mortality was observed with the MP and NP supernatants, respectively, whereas 90–100% cumulative mortality resulted from injection of supernatants prepared from all four HP strains (Fig. 4). Thus, exotoxicity appears to be an HP-specific phenotype, and may be useful for future classification of new *V. nigripulchritudo* isolates. Among SFn1-derived strains, only VN157 demonstrated notable exotoxicity. Nearly 100% of shrimp injected with supernatant from this strain died within 24 h, as was observed using supernatant from wt SFn1. In contrast, strains containing only a pB1067-derived plasmid induced mortality in 0–20% of shrimp. These data indicate that pA1066 is necessary and sufficient to induce supernatant toxicity.

To gain insight into the nature of the toxic factor, SFn1 supernatants were either heat-treated at 85°C for 15 min or size-fractionated by filtration (cut-off of 10 or 50 kDa) prior to shrimp injection. Only untreated crude extracts induced mortality, suggesting that one or more proteinaceous compounds with a molecular weight > 50 kD might be implicated in toxicity (not shown). SFn1 supernatant treated with protease inhibitors (10 mM EDTA or 10 mM 1, 10-phenanthroline) had an effect on shrimp equal to that of untreated supernatant, suggesting that metalloprotease-like enzymes, such as those produced by *V. splendidus* (Le Roux *et al*., 2007) and *V. tubiashi* (Hasegawa *et al*., 2008) that induce damage in Pacific oysters, are not pivotal for *V. nigripulchritudo* pathogenicity. Finally, culture supernatants were analysed using SDS-PAGE, in an attempt to identify toxin candidates; however, the complexity of these supernatants prevented identification of virulence-linked differences (data not shown).

## Discussion

The virulence of *V. nigripulchritudo* towards the shrimp *L. stylirostris* has been previously correlated with the presence of a small plasmid, pB1067, and a few chromosomal markers (Reynaud *et al*., 2008). In this study, we report that a second plasmid, pA1066, identified during genomic sequencing of an HP *V. nigripulchritudo* isolate, can also serve as a marker of the HP phenotype. A variant of this plasmid is present in MP strains (pA1066 type MP), suggesting that a precursor of pA1066 was acquired by the common ancestor of MP and HP strains; however, these plasmids have diverged, as they can readily be distinguished by PCR analyses. Notably, we demonstrate that pA1066 is critical for the full virulence of HP *V. nigripulchritudo* in several experimental models of infection. Plasmid pB1067 also contributes to virulence; in most models, only strains containing both plasmids induced the highest level of shrimp mortality. Thus, the emergence of strains of *V. nigripulchritudo* that are highly pathogenic for *L. stylirostris* appears to depend on the presence of two plasmids, pB1067 and a distinct form of pA1066, and is correlated with several previously described chromosomal polymorphisms.

Understanding the nature of pA1066's contribution to *V. nigripulchritudo* virulence should provide significant insight into the disease process induced in shrimp by this bacterium. However, analysis of this question has been significantly complicated by the difficulty of performing genetic manipulations on this plasmid. We have attempted to disrupt numerous genes within this replicon; however, all suicide vectors that we have tested appear to be incompatible with pA1066, as no co-integrants could be obtained. Consequently, it has not been possible to disrupt candidate virulence genes or the many genes of unknown function within this replicon, to address the significance of the encoded proteins in *V. nigripulchritudo* pathogenicity, or to assess whether pA1066 is a conjugative plasmid. This apparent incompatibility also complicated our analysis of the role of the co-resident plasmid, pB1067, as suicide vector integration within pB1067 typically results in the loss of pA1066. In future studies, we will explore whether non-R6K-based vectors or chitin-based natural transformation may enable genetic manipulation of pA1066 (Meibom *et al*., 2005; Gulig *et al*., 2009). Once mutations can be generated, it will be possible to investigate the function of numerous features of this large plasmid. For instance, we can explore if pA1066 is a self-transmissible conjugative plasmid. Plasmid pA1066 might also enable transmission of other, non-conjugative plasmids, such as pB1067, as has been observed for conjugative plasmids in *V. vulnificus* (Lee *et al*., 2008) and *V. fischeri* (Dunn *et al*., 2005). Transmission of both pA1066 and pB1067 could potentially promote development of new pathogenic strains*.* Conjugation/secretion systems have also been found to enable the secretion of virulence proteins directly into host cells (e.g. in *Bordetella pertussis* and *Helicobacter pylori*; Christie and Vogel, 2000); thus, it is also possible that these pA1066 genes directly contribute to virulence.

One gene cluster within pA1066 whose contribution to virulence clearly warrants investigation encodes a putative MARTX toxin and its transporter. Such toxins have been associated with virulence of *V. cholerae* (Lin *et al*., 1999), *V. vulnificus* (Liu *et al*., 2007) and *V. anguillarum* (Li *et al*., 2008), and they have been identified in several other pathogens as well (Satchell, 2007). As a secreted toxin, MARTX could be linked to the mortality that results from injecting shrimp with *V. nigripulchritudo* culture supernatants, particularly since the active factor appears to be a protein of > 50 kD. Arguing against this possibility is the fact that supernatants from MP strains, which also contain a MARTX gene cluster (within pA1066 type MP), do not induce mortality. However, it is possible that protein expression or secretion differs between the HP and MP strains or that this toxin acts in concert with additional, HP-specific factors. The potential toxin domains encoded within the pA1066 MARTX cluster include a Rho GTPase inactivation domain (RID), an adenylate cyclase domain (ExoY) and two domains of unknown function. Rho GTPases have previously been shown to induce cell rounding; we used previously developed cell culture-based assays (Binesse *et al*., 2008) to determine whether supernatants from various *V. nigripulchritudo* strains also induced this effect. However, supernatants from HP, MP and NP strains all induced a significant cytopathic effect, including cell rounding, perhaps due to the presence of abundant proteases within these supernatants (C. Delsert, pers. com). Consequently, it was not possible to ascertain whether MARTX-linked changes in cell morphology occurred, as such changes could easily be masked by changes induced by other factors produced by all strains.

In this study we supplemented our previously used model of virulence (intramuscular injection of bacteria) with two additional assays to evaluate the pathogenicity of *V. nigripulchritudo*. The non-invasive immersion model mimics the natural infection process, but is quite laborious, while the injection of supernatants enables rapid and reproducible screening. The results from these assays were congruent in several aspects, particularly in demonstrating that pA1066 is required for virulence in all three models, and thus that pB1067, while present in the pathogenic isolates, is not sufficient for their virulence. However, the differences among them suggest that each assay may enable dissection of discrete aspects of the virulence process, for example concerning the role of pB1067. In the immersion model, only strains containing both pA1066 and pB1067 were virulent, while in the bacterial injection model, SFn1, which contains both plasmids, was more virulent than a strain containing pA1066 alone. When supernatants were injected, toxicity was independent of the presence of pB1067. Collectively, these findings suggest that there may be interactions between factors encoded on the two plasmids. It is likely that differences between results obtained with the models reflect the different host-imposed barriers that are encountered by the bacteria. They may also indicate that there are multiple pathways by which the bacterium impairs shrimp viability.

Multilocus sequence typing analyses have demonstrated that HP strains, along with MP strains, differ at several chromosomal loci from NP strains, and that they comprise a monophyletic clade that is evolutionarily quite distant from the NP isolates (Goarant *et al*., 2006a). Subsequent SSH analyses identified a small plasmid whose presence was limited to HP isolates, and several chromosomal loci whose presence largely correlated with the HP phenotype (Reynaud *et al*., 2008). Here, we have found that HP and MP strains both contain large plasmids that share a common origin, based on the sequence identity of their *rep* gene (Fig. 2) and other loci (not shown). Related plasmids were only rarely detected among NP isolates, and they formed a distinct evolutionary unit, congruent with the MLST data. PCR analyses suggest that the pA1066 form HP plasmid may contain sequences that are absent from the MP form. Collectively, these results suggest that a pA1066 ancestor was the first of the three virulence-linked markers to be acquired. Subsequently, the current HP strains acquired pB1067, the HP-specific modification of pA1066, and the chromosomal polymorphisms; however, the relative order of these events cannot be determined, due to the absence of strains containing only a subset of these HP-specific features within our collection.

The absence of such intermediate forms suggests several interesting possibilities regarding the evolution of HP strains. First, such strains may have arisen relatively recently, which would be consistent with the lack of sequence polymorphisms in pA1066 among the various isolates. Additionally, the apparent temporal coupling of pB1067 acquisition and modifications of pA1066 and the chromosome suggests that these events may be interconnected, i.e. that changes in one locus prompted or provided a selective force for subsequent changes. Presumably the combination of changes that led to the emergence of the HP lineage has rendered it particularly suited to a niche or habitat.

It remains to be seen whether chromosomal polymorphisms, like the two HP-specific plasmids, are required for full virulence; it is possible that they serve as markers of this lineage without directly contributing to pathogenicity. Still, it seems clear that the pathogenic phenotype is a feature of a very specific subset of strains with largely uniform genetic content. Comparisons of the complete genomes of several HP, MP and NP organisms, along with the development of new genetic tools for engineering of *V. nigripulchritudo*, will yield insight into the key features underlying virulence, and facilitate exploration of whether horizontal gene acquisitions have driven the differentiation of this virulent lineage.

## Experimental procedures

### Bacterial strains, plasmids and culture conditions

*Escherichia coli* strains DH5αλpir and β2163 (Demarre *et al*., 2005) were used for cloning and conjugation respectively. The *V. nigripulchritudo* unmodified isolates have been described previously (Goarant *et al*., 2006b). SFn1 derivatives and plasmids used in this study are described in Tables 2 and 3. *Vibrio nigripulchritudo* strains were grown in LB + NaCl 0.5 M, marine broth (MB) 1× or 5×, or on marine agar (MA) at 30°C. *Escherichia coli* strains were grown in Luria–Bertani (LB) at 37°C. Matings between *E. coli* and *V. nigripulchritudo* were performed at 30°C as described previously (Le Roux *et al*., 2007). Spectinomycin was used at 100 μg ml^−1^. Diaminopimelate (DAP) was supplemented when necessary to a final concentration of 0.3 mM.

### Molecular biology techniques

PCR was performed using Pfu Ultra DNA polymerase (Stratagene) or Hot Start DNA polymerase (Qiagen), according to the manufacturer's instructions. Primers are listed in Table S1. Plasmid DNA was isolated using a spin miniprep kit (Qiagen).

### Plasmid construction

A fragment of pB1067 that enables autonomous replication of a suicide vector (F. Le Roux, B.M. Davis and M.K. Waldor, in preparation) was amplified with primers pB2700f and pB3400r then cloned into BamHI site of pSW25T, yielding pSW110. Nucleotides 2000–2530 of pB1067 were amplified with primers pB2000f and pB2530r. The resulting product was digested with BamHI and ligated to pSW25T, yielding pSW68. Nucleotides 28–480 of pB1067 were amplified with primers pB28f and pB480r, then similarly digested and ligated to yield pSW120. The results of all ligations were confirmed by plasmid sequencing using primers SW25seqS and AS.

### Strain construction

Plasmids were conjugated from β2163 into various *V. nigripulchritudo* strains. To generate strains VN110 and VN157, 10 Spec^R^ exconjugants generated following transfer of pSW110 to SFn1 were restreaked, then scored by PCR or visualization of purified plasmid DNA for the presence of pSW110, pA1066 and pB1067, using primers SW25seqS and SW25seqAS, pA1f and pA1r or pB28f and pB480r respectively. Of these 10 Spec^R^ colonies, nine lacked pA1066 and pB1067, and one of these was named VN110. The single Spec^R^ isolate that lacked pB1067 but contained pA1066 was cultivated in LB/NaCl without antibiotic selection. After two subcultures (1/50), a Spec^S^ clone that had lost pSW110 but maintained pA1066 was identified. No loss of pA1066 from this strain (VN157) was observed following growth of additional subcultures.

Spec^R^ exconjugants generated by transfer of pSW68 and pSW120 into SFn1 were scored, using PCR, for recombination of each plasmid with the appropriate site in pB1067 and for the presence of pA1066. All exconjugants were found to lack pA1066. Transfer of pSW68 yielded VN68, and transfer of pSW120 yielded VN120.

### Experimental challenge

*Vibrio nigripulchritudo* strains and mutants were cultured in MB for 18 h at 30°C with constant shaking, yielding late exponential phase cultures. Bacterial concentrations in these cultures were determined by comparing their optical density at 600 nm to a previously established reference curve. Experimental infections in *L. stylirostris* shrimp were performed either by immersion or by intramuscular injection of bacterial cell suspensions or supernatants between the third and fourth abdominal segments.

For the injection challenge, 50–500 cfu were intramuscularly injected into healthy shrimp kept in aquaria (2 replicate aquaria with 10–20 shrimp in each for each strain) filled with filtered seawater, aerated and held at 27°C. Control shrimp were injected with an equal volume of sterile culture medium. Survival was monitored daily over a 3-day period, because preliminary trials demonstrated that no significant mortality occurred after this time.

Bacterial supernatants were prepared from bacterial cultures grown in MB 5× as described previously (Goarant *et al*., 2000). When indicated, crude supernatants were either denaturated by heat treatment at 85°C for 15 min or size-fractionated by filtration through Centricon YM-10 and YM-50 ultrafiltration units (Millipore, molecule weight cut-off, 10 and 50 kDa respectively). Animals were injected with 100 μl of each preparation or control medium. Experiments were conducted in duplicate (8–12 shrimp/aquarium). Toxicity was assessed after 24 h.

Immersion challenges were performed in 100 l of filtered and aerated seawater containing 10^5^ cfu ml^−1^ of the considered vibrio strain (Saulnier *et al*., 2000). Following a 2 h challenge, 10–20 individuals were transferred to 100 l tanks filled with filtered seawater, aerated and held at 27°C. Control shrimp were treated as above except that bacterial cultures were not added during exposure periods. Three replicate tanks were used for each treatment. For each type of challenge, experiments were repeated twice. Two and 24 h after infection, four shrimp from each experimental condition were randomly sampled. Haemolymph was collected using a 25-gauge needle containing an anticoagulant solution (2% NaCl, 0.1 M glucose, 30 mM sodium citrate, 26 mM citratic acid, 10 mM EDTA) and serial dilutions were plated onto solid MA with 2% glycerol (w/v) and incubated at 30°C for 72 h. Putative *V. nigripulchritudo* colonies were identified as black colonies.

### *In silico* analysis

The annotation of pA1066 has been performed using the MaGe software [Magnifying Genome (Vallenet *et al*., 2006)] (https://www.genoscope.cns.fr/agc/microscope/mage/).

The *rep* sequences were aligned using Seaview and phylogenetic trees were built using Phylo-win program (Galtier *et al*., 1996) applied to Neighbour-joining method and Kimura's two-parameter distances. Reliability of topologies was assessed by the bootstrap method with 1000 replicates. The plasmid map of pA1066 (Fig. 1) was generated using the BioPython and GenomeDiagram (Pritchard *et al*., 2006) open software packages.

## Notes

**Fig. S1.** The replication/segregation module. **Fig. S2.** The MARTX (multifunctional autoprocessing repeats in toxin) gene cluster (A) and domain annotation (B). **Fig. S3.** The putative conjugative machinery. This cluster includes homologues of the core proteins of a type IV secretion system (in green), the coupling protein TraD and a relaxase-helicase TraI (yellow and orange). **Table S1.** Oligonucleotides used in this study.
