# Supplementary material for: Virulence of an emerging pathogenic lineage of Vibrio nigripulchritudo is dependent on two plasmids
Source: Environ Microbiol. 2010 Sep 6;13(2):296–306. doi: 10.1111/j.1462-2920.2010.02329.x (PMC3020234; doi:10.1111/j.1462-2920.2010.02329.x)
Supplement: Supplementary file 1 — Fig. S1. The replication/segregation module. Fig. S2. The MARTX (multifunctional autoprocessing repeats in toxin) gene cluster (A) and domain annotation (B). Fig. S3. The putative conjugative machinery. This cluster includes homologues of the core proteins of a type IV secretion system (in green), the coupling protein TraD and a relaxase-helicase TraI (yellow and orange). Table S1. Oligonucleotides used in this study. [file emi0013-0296-sd1.doc]

Supplementary data

Table S1

Oligonucleotides used in this study

| Oligonucleotide | Sequence (5’-3’) |
| --- | --- |
| SW25seqS | cggtatcgataagcttgatatc |
| SW25seqAS | ggcggccgctctagaactagt |
| pB2700f | GCCCGGATCCCGCGCTATCGCTTGTCGGCC |
| pB3400r | GCCCGGATCCAAGAGCAGGGCTTTATATAGGACG |
| pB2000f | GCCCGGATCCGGACAGGATTTAACATTGAGGGG |
| pB2530r | GCCCGGATCCGAAAGGGCACCCAATAGGGTGC |
| pB28f | gcccggatccTTGTTCGTGTTGAGAATAGC |
| pB480r | gcccggatccCAGCATTTAAGGCCTTACTC |
| pA1f | TTGGCTATCATTGCTGGTGCA |
| pA1r | TGTCTTCGATGGGACTGAACAA |
| pA2f | ATGCAGAAGCCTGGCGGGA |
| pA2r | GGAGCAGAGACATTAAGTCGGT |
| pA3f | TCCCGTGTCCCATGCGTTCCAT |
| pA3r | ACGCAAACGCAGAAGGCAATGT |
| pA4f | CGTAGGGTTCCTCAGAGAATTCT |
| pA4r | GCATTGCTCCCATATTCGCAGG |
| pA5f | GGCAGGTTGGTCAAAGCGGTCT |
| pA5r | TGAGTAAGCAGTGGCAGCGTAC |
| pA6f | GTCTCGGGTTTCTTCTGGTTCA |
| pA6r | TGGAACGACGATGAACTCGCCT |
| pA7f | CTAACAAGCCAGCTCCAGGT |
| pA7r | ACCAAAGTCAGACAATCCTCTG |
| pA8f | AGGACGTTCTGGTGGTGGGCTA |
| pA8r | CTGAAACAATGCGTAGCGATAG |
| pA9f | CTTTGAGTCCCATAACCCCCGT |
| pA9r | TCATAGAATCGTTCCCACTGGT |
| pA10f | CAACACGGGAGCTGATGCTGT |
| pA10r | CATAGTCTGATTATTGAGCGT |

**Figure S1**

The replication/segregation module.

**Figure S2**

The MARTX (multifunctional autoprocessing repeats in toxin) gene cluster (A) and domain annotation (B).

**Figure S3**

The putative conjugative machinery. This cluster includes homologs of the core proteins of a Type IV secretion system (in green), the coupling protein TraD and a relaxase-helicase TraI (yellow and orange).

**Figure S1**

**
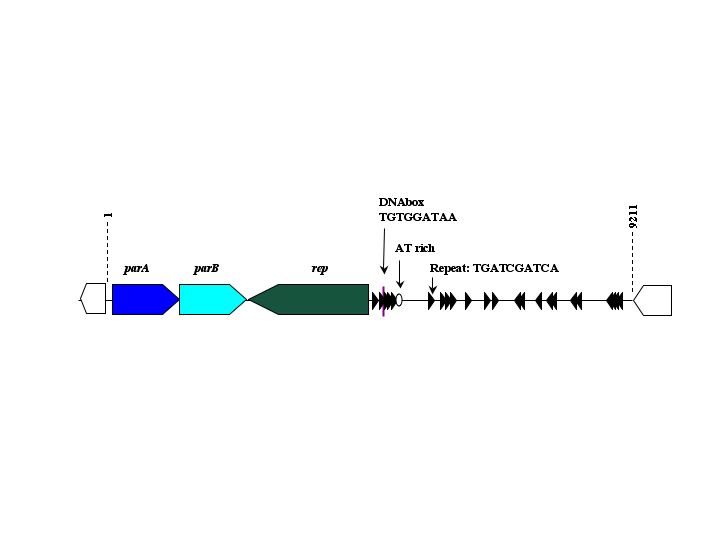
**

**Figure S2**

**
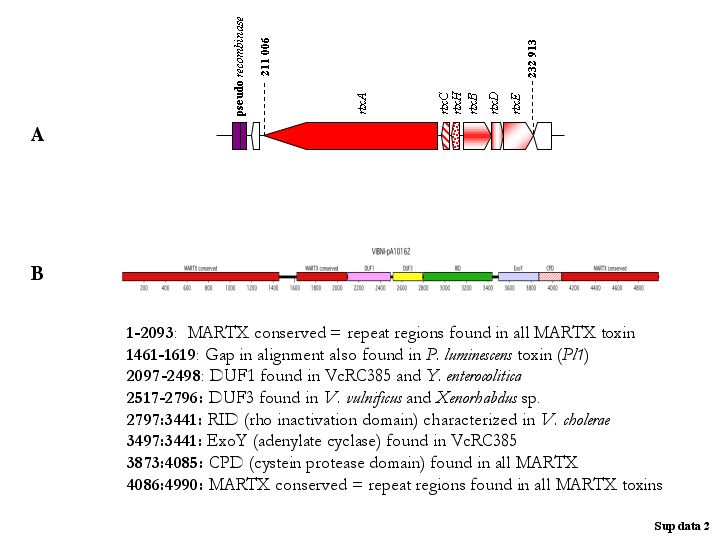
**

**Figure S3**

**
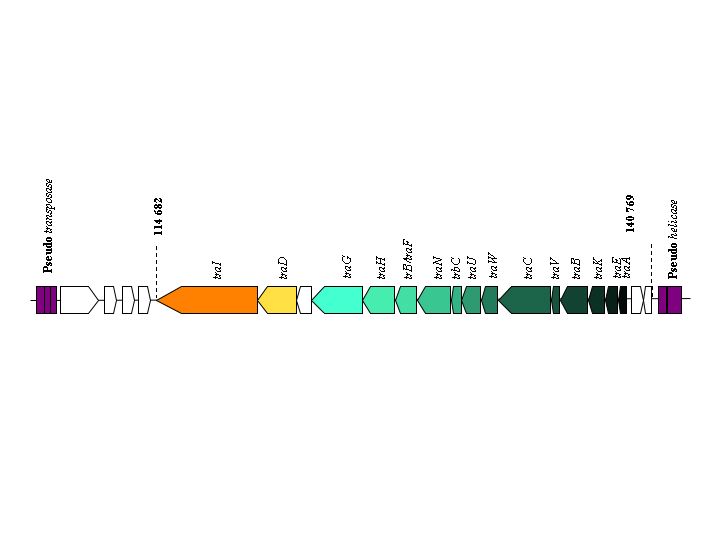
**
